# The Therapeutic Benefits of Single and Multi-Strain Probiotics on Mean Daily Crying Time and Key Inflammatory Markers in Infantile Colic

**DOI:** 10.7759/cureus.28363

**Published:** 2022-08-24

**Authors:** Jenna M Sheldon, Noel Alonso

**Affiliations:** 1 Pediatrics, Nova Southeastern University Dr. Kiran C. Patel College of Osteopathic Medicine, Davie, USA; 2 Pediatrics and Emergency Medicine, Nova Southeastern University Dr. Kiran C. Patel College of Osteopathic Medicine, Davie, USA

**Keywords:** lactobacillus, probiotics, microbial dysbiosis, inflammatory cytokines, human microbiome, infantile colic

## Abstract

Infantile colic is a functional gastrointestinal disorder in which a healthy infant displays paroxysms of intense crying or fussiness. Although this condition is self-limited, it causes significant distress for parents and may be linked to long-term health concerns for children. The microbiome of infants with colic has been correlated with increased dysbiosis or imbalance of commensal bacteria. This dysbiosis may ultimately lead to changes in infants’ immunological profiles, favoring markers linked to inflammation, including specific cytokines, calprotectin, and genetic markers. Therapeutic regimens such as probiotics may be helpful in modifying the gut microbial composition, thereby influencing the presence of inflammatory markers and potentially reducing colic symptoms in infants. This review provides a summary of the findings from 10 randomized, placebo-controlled, double-blinded studies conducted in the past five years with the aim of examining the potential therapeutic benefits of probiotics in infantile colic. The articles were selected through PubMed and Google Scholar using the keywords infantile colic, microbiome, probiotics, cytokines, dysbiosis, inflammatory markers, and lactobacilli. We summarize the results of these studies to explore the potential anti-inflammatory therapeutic benefits of single and multi-strain probiotic formulations on daily crying time and key inflammatory markers in infants with colic. The research largely shows the beneficial role of probiotics, largely of the lactobacillus genus, in the reduction of colic symptoms and the reduction of key inflammatory markers. However, some studies demonstrated an insignificant effect of certain probiotic strains in symptom management. Further research is necessary to better understand the anti-inflammatory properties of probiotics and determine the role this could have on the manifestation of colic in infants.

## Introduction and background

Infantile colic is a condition of excessive crying and distress in otherwise healthy babies. The definition of colic varies within the existing literature but is currently primarily defined using either Rome IV criteria or Wessel criteria [1,2]. The Rome IV criteria require the infant to be less than five months of age when the colic symptoms start and stop. It defines colic as “recurrent and prolonged periods of infant crying, fussing, or irritability reported by caregivers that occur without obvious cause and cannot be prevented or resolved by caregivers.” Lastly, the Rome IV criteria qualify that no evidence of infant failure to thrive, fever, or illness may be present when diagnosing infantile colic [2]. The Wessel criteria define colic as crying and/or fussing episodes lasting for three or more hours per day, for three or more days per week, and for three or more weeks [1]. However, for practical reasons, researchers tend to rely upon the modified Wessel criteria in a clinical setting, defined as crying and/or fussing for three or more hours per day, for three or more days per week, and for one week [2].

The prevalence of colic varies based on age and criteria used, with the highest prevalence occurring in the first six weeks of life [3]. Many studies have examined associated risk factors, treatments, and clinical outcomes with colic patients [4-7]. However, infantile colic poses several challenges for examination including those associated with long-term assessment in clinical settings, subjective reporting measures, and an inability to control for certain independent variables. Additionally, colic is a self-limited condition, so it is important to consider whether results are attributed to treatment measures or a natural resolution of symptoms over time. It is important to better understand colic as this condition may result in parental distress, resulting in a higher incidence of non-accidental trauma and an increase in doctor visits due to heightened concerns. Several studies suggest long-term consequences for children with crying and feeding problems [8,9].

Previous studies have established a link between gut microbiome dysbiosis and infantile colic. Specifically, coliform strains are more prevalent in infants with colic compared to healthy infants without colic [10,11]. Recent research utilizes this known association between the gut microbiome and colic to assess for potential implications of treatment with agents that modify the microbiome. Probiotics, for example, are commonly relied upon to modify the microbiome due to their beneficial effects, including their anti-inflammatory properties. Certain strains of *Bifidobacterium* and *Lactobacillus* have shown utility in reducing C-reactive protein (CRP) levels in inflammatory disorders and reducing inflammatory markers such as interleukin (IL)-6 and tumor necrosis factor (TNF)-α in adult populations [7]. Both IL-6 and TNF-α are two important factors responsible for signaling events that lead to acute inflammation, including the induction of CRP. Previous studies have suggested an association of colic with low-grade systemic inflammation, which may be due to the alteration of gut microbiota composition and inflammatory markers [12]. To better understand the role probiotics may play in reducing inflammation associated with colic, several studies have examined the fecal microbiota composition, metabolomic composition, fecal markers, and blood immune markers after the administration of probiotics. We note a recent review conducted by Skonieczna-Żydecka et al. [13] in 2020 examining the effect of probiotics on symptoms, gut microbiota, and inflammatory markers in infantile colic. This meta-analysis included studies from their respective databases’ inception leading up until December 2020. This current review aims to further contribute by examining studies that meet our inclusion criteria within the past five years. In an effort to better understand potential therapeutic options for colic, this literature review further examines the results from 10 recent studies with the general aim of determining the role of probiotics in infants with colic in terms of potential for reduction in colic symptoms and/or reduction of inflammatory markers.

## Review

Articles were identified through PubMed and Google Scholar using the following keywords: infantile colic, microbiome, probiotics, cytokines, dysbiosis, inflammatory markers, and lactobacilli. All studies were required to be randomized, double-blinded, and placebo-controlled studies published within the last five years from the date this review was conducted. All studies were reviewed to ensure subjects were less than five months of age to ensure that they qualified for the ROME IV criteria of colic, which requires an infant to be less than five months of age when their colic symptoms start and stop. We also selected studies based on criteria for the methods used, including implementation of probiotic treatment with 21-42 days of treatment and utilization of validated assessments for measuring infants’ colic symptoms. Two investigators independently reviewed the included studies to confirm their eligibility for inclusion based on the proposed inclusion criteria. In total, we identified 10 publications that met the criteria to be included in the review.

*Lactobacillus reuteri* (DSM) 17938

*Lactobacillus reuteri *colonizes the gastrointestinal tract, urinary tract, skin, and breast milk of humans. Recent studies established a potential anti-inflammatory role for *L. reuteri*, including inhibiting inflammatory mediators, eicosanoids, and pro-inflammatory cytokines [9]. Three of the studies that met the inclusion criteria for this review utilized* L. reuteri *as a single-stranded probiotic treatment intervention [14-16]. The results from these three studies included 315 total children, 169 of which received the treatment intervention of *L. reuteri* (doses ranging from 1×10^8^ to 5×10^8^) and 146 received a placebo intervention. All three studies examined mean daily crying time throughout the course of treatment. However, each study provided the therapeutic intervention for a unique set of times, including 28, 30, and 42 days. All infants were either exclusively or predominantly breastfed, with the exception of the subjects who took part in the 28-day intervention, which included formula-fed infants only (n=124 for the treatment group and n=117 for the control group). Overall, the infants who received *L. reuteri *or placebo for the studies with treatment timelines of 28 and 42 days did not show a significant reduction in crying time of the treatment groups (n=137) compared to the control groups (n=124) [14,16]. In fact, the results of the 28-day intervention study actually showed a significant reduction in the crying time for the placebo group (n=117) compared to the treatment group (n=124) (p=0.001) [16]. Conversely, the results for the *L. reuteri* therapeutic intervention lasting 30 days demonstrated a significant reduction in daily crying time between the control group (n=22) and the treatment group (n=32) (p=0.001) [15]. At the end of the 30 days, the control group demonstrated an average crying time of 147.85±37.99 minutes per day compared to the treatment group, which demonstrated 74.67±25.04 minutes per day.

In addition to examining mean daily crying times, two of the three studies examined key inflammatory markers present in the treatment and control groups at the end of the intervention with *L. reuteri *DSM 17938 [14,15]. The results for the 42-day intervention showed a nonsignificant difference between the treatment group (n=11) and the control group (n=5) for the percentage of FOXP3+Tregs, cells that play an important role in regulating immunological processes in the gut, in peripheral blood. However, the plasma IL-2 level was also significantly lower in the treatment group at the end of the 42 days (p=0.05) [14]. Subsequent data collected by Savino et al. [15] in the 30-day intervention study showed a significant increase in FOXP3 concentrations (p=0.009), a decrease in RORγ/FOXP3 ratio (p=0.023), and reduced fecal calprotectin (p=0.001) in the *L. reuteri* treatment group (n=32) compared to the control group (n=22) at the conclusion of the 30 days. 

*Lactobacillus rhamnosus* GG (ATCC 53103)

*Lactobacillus rhamnosus *GG is a bacterial species commonly found naturally in the digestive tract and within vaginal flora as well. This bacterial strain has been shown to exert an anti-inflammatory effect on epithelial cells, which makes it of particular interest to study in conditions such as colic, which may have an inflammatory etiology [17]. One of the 10 selected studies [18] examined *L. rhamnosus* (ATCC 53103) as a single-stranded probiotic to determine the role it plays in influencing immunological biomarkers and intestinal microbiota composition in infants with colic. This study by Savino et al. [18] examined the role of *L. rhamnosus* in combination with cow milk avoidance in the management of colicky breastfed infants. The study included 24 infants in the treatment group who were given *L. rhamnosus* at a concentration of 5×10^9 ^and 21 infants in the control group. The* L. rhamnosus* group showed a significant reduction in crying from day 0 (242 minutes) and day 28 (104.7 minutes) of treatment (p=0.001). On the other hand, the placebo group showed a nonsignificant reduction between day 0 (247.9 minutes) and day 28 (239.6 minutes) of treatment (p>0.05).

Savino et al. [18] also performed a microbiological analysis of fecal samples from participants. There was a significant increase in total bacteria within the fecal samples (p=0.04), including a significant increase in fecal *Lactobacillus* spp. after 28 days of treatment (p=0.0483). However, the study did not find a significant difference in the amount of *E. coli *found in the samples at the end of the treatment course. The levels of the inflammatory marker, calprotectin, were also examined in the fecal samples. Fecal calprotectin levels showed a significant decrease in the treatment group of -119.53 (p<0.05) compared to the nonsignificant change in the placebo group of -10.41 (p>0.05) at the end of the 28 days.

*Bifidobacterium animalis *subsp. *lactis*

*Bifidobacterium animalis *is a gram-positive anaerobic bacterium that naturally colonizes the gut of humans. Three of the studies that met the inclusion criteria for this review examined this single-strain probiotic in the treatment group [19-21]. Two of the three studies [19,20] utilized the same concentration of *Bifidobacterium animalis* subsp. *lactis *of 1×10^9^ CFU/day. A total of 136 infants were examined in the treatment groups, and 136 infants were included in the control groups. One of the studies provided the probiotic intervention for 21 days [19], and the other study provided the intervention for 28 days [20]. Both studies showed a significant decrease in the average crying duration in the treatment group compared to the control. The 21-day intervention study showed a mean daily crying time of 60.8±23.4 minutes in the treatment group compared to the control group, which had an average of 95.8±26 minutes at the end of the intervention (p<0.0001). The results from the 28-day intervention demonstrated a reduction in crying time from 129.9±43.7 minutes per day in the control group to 84.3±51.4 minutes per day in the treatment group (p=0.001).

In addition to examining mean crying time, Nocerino et al. [20] also examined the levels of key innate and acquired immune markers in fecal samples of both study groups. The sample size for this data was reduced due to limitations in the number of fecal samples available, including a sample size of n=32 in the treatment group and n=30 in the control group (originally n=40 for both treatment and control groups for the primary endpoint of reduced crying time). The inflammatory markers examined include human β-defensin-2 (HBD-2), cathelicidin (LL-37), secretory IgA (sIgA), butyrate, and calprotectin. A significant increase in HBD-2, LL-37, sIgA, and butyrate was noted in the treatment group (p≤0.05) after the 28-day intervention was complete for both groups. However, unlike the other inflammatory markers, calprotectin showed a decrease in the BB-12 group.

The subsequent study by Chen et al. [19] examined similar markers and also found an increase in sIgA and butyrate at the end of their 21-day intervention. However, this group found an increase in calprotectin in the fecal samples of the BB-12 group compared to the placebo. This study also noted two other increased immune markers, β-defensin-2 and cathelicidin.

A third study by Xinias et al. [21] also examined *Bifidobacterium lactis* BB-12 as an intervention to reduce colic symptoms in infants but included the probiotic in an intervention formula consisting of whey hydrolysate, reduced lactose, *Bifidobacterium lactis *BB-12, and galacto-oligosaccharides for a period of one month (n=40). The control group (n=20) received standard infant formula without the probiotics. The improvement of colic symptoms was tracked via a quality of life (QoL) questionnaire, which assessed measures such as duration of crying and quality of parent-child relationship. The study found a significant reduction in crying time after the end of the month-long intervention for the probiotic group compared to the control group (1.5-hour difference between groups) (p<0.001). Additionally, the data demonstrated a significant improvement in the parent-rated quality of parent-child relationship at the conclusion of the study for the probiotic group, but not for the control group.

Multi-strain probiotics

Multi-strain probiotics are believed to offer synergistic effects that individual strains may not be able to achieve on their own [22]. Three of the 10 studies included in this review utilized a multi-strain probiotic intervention [23-25].

One of the multi-strain studies that met our inclusion criteria [25] combines two of the aforementioned single-strain probiotics,* L. rhamnosus* and* L. reuteri*. Note that these strains have different designation numbers than the strains utilized in the single-strain studies,* L. rhamnosus* 19070-2 and *L. reuteri* 12246. This study by Gerasimov et al. [25] included exclusively breastfed infants with colic, one of which was given a mixture of the two lactobacillus strains combined with 3.33 mg of fructooligosaccharide (n=84) and 200 IU of vitamin D3, and the other was given vitamin D3 for 28 days (n=84). The researchers of this study measured the crying and fussing time utilizing the validated Baby’s Day Diary and found a significant difference in mean crying and fuss time between the probiotic and control groups, with the control group crying an average of 163 minutes per day and the treatment group crying an average of 116 minutes per day (p=0.019).

A second multi-strain intervention study that met our inclusion criteria was conducted by Baldassarre et al. [23]. Twenty-seven infants were included in the multi-strain probiotic intervention group with a concentration of 5×10^9^ CFUs, and 26 were included in the placebo group with the intervention lasting for 21 days. The multi-strain probiotic used in the intervention group consisted of a lyophilized high-concentration multi-strain probiotic mixture of four different strains of lactobacilli (*L. paracasei *DSM 24733, *L. plantarum* DSM 24730, *L. acidophilus* DSM 24735, and *L. delbrueckii* subsp. *bulgaricus* DSM 24734), three strains of bifidobacteria (*B. longum* DSM 24736, *B. breve* DSM 24732, and *B. infantis* DSM 24737), and one strain of *Streptococcus thermophilus* DSM 24731. The infants in the intervention group demonstrated a statistically significant reduction in the number of minutes crying per day (68.4 minutes per day) compared to the placebo (98.7 minutes per day) (p=0.001).

In addition to examining crying time in both groups, this study also examined fecal samples from both groups to evaluate the microbial content and metabolome utilizing both fecal real-time polymerase chain reaction (qPCR) and nuclear magnetic resonance (NMR)-based analysis. Nineteen fecal samples were evaluated using these techniques (placebo group, n=8; intervention group, n=11). A nonsignificant difference in total probiotic bacteria, *Lactobacilli* and *Bifidobacteria*, was found between the intervention and placebo groups.

A more recent study conducted in 2021 by Chen et al. [24] examined a multi-strain probiotic intervention consisting of* Bifidobacterium longum *CECT7894 and *Pediococcus pentosaceus* CECT8330 at a concentration of 1×10^9^ CFUs. Many studies have previously examined *Lactobacillus* and *Bifidobacterium* in colicky infants, but the genus *Pediococcus* had not yet been examined as extensively in this condition prior to this study. From a phylogenetic perspective, the *Pediococcus* genus is close to the *Lactobacillus* genus. It is also found naturally in breast milk and has been studied previously in a variety of disease states, including obesity, diarrhea, trauma, and *Helicobacter pylori *infection. However, this is the first study examining this combination of probiotic bacteria, *Bifidobacterium longum* and *Pediococcus pentosaceus*, in infants with colic. A total of 48 subjects were included in the intervention group receiving the multi-strain probiotic, and 42 infants were included in the placebo group. The intervention was provided over the course of 21 days with a significant reduction in crying time observed in the intervention group at 7, 14, and 21 days in comparison to the placebo. At the end of the 21-day intervention, the treatment group demonstrated an average crying time of 14 minutes per day compared to the placebo group, which demonstrated an average crying time of 40 minutes per day (p<0.001). Additionally, fecal consistency scores showed a statistically significant improvement in the intervention group compared to the control group on day 21 of intervention only (p<0.001). This study did not examine inflammatory markers as a secondary endpoint.

## Conclusions

In this analysis of 10 randomized, placebo-controlled studies examining probiotics in infantile colic, we found mixed results for both the reduction in crying time and the influence on microbial composition and inflammatory markers. Although the majority of the studies did in fact find a significant reduction in crying time for infants given the probiotic intervention, two of the three studies utilizing single-stranded *L. reuteri* as the probiotic intervention did not show a significant reduction for the treatment groups. Conversely, the third study examining* L. reuteri *and the remaining seven studies examining* L. rhamnosus*, *B. animalis*, and multi-strain interventions all found statistically significant reductions in crying time within the probiotic treatment groups compared to the control groups. It is important to note that one of the two single-stranded *L. reuteri *studies that did not show a significant reduction in crying time for the treatment group only included formula-fed infants in the study, whereas the other two *L. reuteri* studies included infants who were either exclusively or predominantly formula-fed. The seven studies that showed a significant reduction in crying time at the conclusion of the probiotic intervention demonstrated varying differences in the total crying time reduction. Additional research examining the utility of these differences, including whether or not long-term clinical implications are present, would be helpful in understanding the degree of benefit in treating colic.

Although not all studies included the fecal microbial composition or inflammatory markers as endpoints, several of the studies did demonstrate promising results in this regard. Of note, three of four separate studies with interventions involving administration of single probiotics respectively all showed significant reductions in calprotectin within the fecal samples of the treatment group compared to the placebo groups. Furthermore, a small study further showed an increase in FOXP3 in ​​infants given *L. reuteri* over the course of 30 days. These studies suggest a potential role of probiotic strains in reducing the systemic inflammation found in infants with colic.

The limitations of the results of the 10 studies included in this review include the lack of a consensus for a definition of colic within existing literature and the challenges associated with objectively measuring colic symptoms. Colic is a self-resolving condition, so there exists the possibility of natural resolution of symptoms over time with or without therapeutic intervention. Additionally, out of the 10 studies that met our inclusion criteria, seven examined the therapeutic effects of single-strain probiotics and three examined multi-strain probiotics. However, the existing data for both single-strain and multi-strain studies was limited due to the relatively small sample sizes included and the limited number of studies conducted, especially within the past five years. Future analysis further examining both individual and multi-strain probiotics at varying doses with infants diagnosed with colic would provide a more thorough understanding of the role these interventions play in the management of colic. Similarly, additional studies may wish to further examine the impact of including infants who are strictly formula-fed versus strictly breastfed, as diet modification in addition to therapeutic intervention may contribute to differences in findings. Additionally, due to the mixed results in the reduction of crying time and the concentration of inflammatory markers, we recommend repeating similar studies with a larger sample size. Future studies may also benefit from conducting a comprehensive analysis of the microbiome in infants with infantile colic using methods such as full sequencing. This comprehensive analysis may provide a more complete understanding of the etiology and therapeutic treatment options for infantile colic.
